# Evaluation of Antimicrobial Properties and Shear Bond Strength of Conventional Orthodontic Adhesive Modified With Calotropis gigantea Nanoparticles: An In Vitro Study

**DOI:** 10.7759/cureus.51182

**Published:** 2023-12-27

**Authors:** Divya Ravuru, Ganugapanta Vivek Reddy, Arun Bhupathi, Karumuri Taraka Sunil Kumar, Gowri sankar Singaraju, Prasad Mandava

**Affiliations:** 1 Orthodontics, Oxford Dental College, Bengaluru, IND; 2 Orthodontics and Dentofacial Orthopaedics, Narayana Dental College, Nellore, IND; 3 Orthodontics, Vishnu Dental College, Bhimavaram, IND; 4 Pharmaceutical Quality Assurance, Shri Vishnu College of Pharmacy, Bhimavaram, IND

**Keywords:** orthodontic, nanoparticles, calotropis gigantea, bond strength, antimicrobial, adhesive

## Abstract

Introduction: Bonding of brackets with adhesives during orthodontic fixed appliance therapy is associated with white spot lesions (WSLs). An adhesive developed with an antimicrobial property is advantageous to prevent decalcification of the enamel surface. The current study assesses the antimicrobial and shear bond strength (SBS) characteristics of an experimental conventional orthodontic adhesive incorporated with different concentrations of nanoparticles (NPs) prepared from the leaves of *Calotropis gigantea* and compares them with non-admixed conventional adhesive.

Materials and methods: A total of 40 premolar teeth therapeutically extracted for orthodontic purposes were randomly assigned to four equal groups of n = 10 each. In control Group I, unmodified conventional adhesive was used to bond the brackets. In the three experimental groups, Group II, Group III, and Group IV, the brackets were bonded with modified conventional adhesive admixed with the *C. gigantea* NPs in concentrations of 1%, 5%, and 10%, respectively. The agar well diffusion test and the disc agar diffusion (DAD) test were utilized for assessing the anti-microbial activity of the composite discs. The SBS of the groups was determined by a universal testing machine.

Statistical analysis: A one-way ANOVA and Tukey’s honestly significant difference (HSD) post-hoc test was used to analyze the difference in shear bond strength and microbial inhibition zone diameter between the groups with a probability (p) value of equal to or less than 0.05 as statistical significance.

Results: The mean SBS for Group I without nanoparticles is 6.99±0.75 MPa. The SBS value decreased inversely in proportion to the concentration of NPs: Group II (1% NP) 6.29±0.67 MPa; Group III (5% NP) 4.40±0.47 MPa; and Group IV (10% NP) 1.98±0.21 MPa, which is statistically significant (p < 0.001). The incorporation of *C. gigantea *NPs resulted in a decrease in the actual microbial potency of the conventional adhesive.

Conclusion: Isolated *C. gigantea* NPs, when used alone, proved to have antimicrobial efficacy, but orthodontic adhesives admixed with *C. gigantea* NPs showed no additive effect, and SBS values decreased with increased concentrations of NPs.

## Introduction

In orthodontics, resin-based composites have primarily been utilized as adhesive agents to bond the brackets to the tooth surface. These have undoubtedly remained the first option for most orthodontists for bonding various attachments besides brackets, ever since they were introduced to the orthodontics field. The technique of orthodontic bonding is highly advantageous in that it is simple and highly esthetic. At the same time, it carries the risk of increased plaque accumulation and, hence, the occurrence of demineralization areas around the bracket known as white spot lesions (WSLs) [1].

Typically, orthodontic brackets and other appliances complicate routine oral hygiene procedures and increase bacterial plaque accumulation, resulting in enamel demineralization. The cariogenic bacteria, particularly *Streptococcus mutans* and *Lactobacillus acidophilus*, increase significantly during orthodontic treatment. These bacteria synthesize organic acids at a pH level of 5.5, causing WSLs and subsequent dental caries. At some point in time, remineralization is not possible in these lesions and is a major concern for both the patient and the orthodontist [2].

The first preventive measure for preventing WSLs is maintaining good oral hygiene, which includes using fluoride toothpaste and brushing teeth; however, it relies entirely on the compliance of the patients. Dentistry and medical literature have extensively documented the issue of poor patient compliance, and less than 15% of orthodontic patients follow instructions regarding hygiene, according to Geiger et al. [3]. As a result, the scientific community has focused on orthodontic biomaterials like adhesives, ligatures, brackets, etc. that have inbuilt antibacterial or anti-cariogenic potential. Plaque accumulation around composite adhesives used for bonding brackets may increase the chance of demineralization of the enamel [1,4]. Therefore, if a bonding agent were to be made antibacterial, it may inhibit bacterial growth and prevent demineralization near the enamel surface of orthodontic brackets. The use of such bonding systems with inherent antimicrobials around the brackets may be beneficial to non-compliant patients.

So, in an attempt to reduce the incidence of WSLs, many manufacturers have been in search of novel methods and materials with anti-cariogenic properties. Among the substances that have attracted a lot of interest lately for their antibacterial properties are nanoparticles (NPs), especially metal NPs. Nanoparticles are insoluble particles that are less than 100 nm in size. When compared to non-nanoscale particles, they have a high surface area to volume ratio that improves their contact with bacterial cell membranes and offers a noticeably bigger surface area for antimicrobial activity [5].

Several studies have evaluated the antimicrobial efficacy of various metal NPs [6-9] and plant extracts [10-12] incorporated into orthodontic composite resins. The use of synthetic materials (metal NPs) causes the release of metal ions, which can exhibit cytotoxicity risks [5]. Finding natural products with antiplaque and antimicrobial properties that replace metal ions could be very beneficial in such cases. The utilization of natural products has become more popular, and active plant extracts are frequently employed in the synthesis of NPs. One such plant extract that has been recently used is *Calotropis gigantea *which is a traditional medicinal plant. In a study by Jim et al. [13], the phytochemical research revealed that the leaf and root extracts of this plant contain flavonoids, alkaloids, steroids, terpenoids, tannins, and phenols, which may possess antibacterial action. In leaves, anthraquinone is present, and leaves have more potential than roots. Incorporating NPs into composite resins has the potential to modify their mechanical and physical properties in addition to causing certain biological and chemical alterations [14]. Very sparse literature is found regarding the use of *C. gigantea*-coated NPs in dentistry and specifically in orthodontics. Therefore, this in vitro study aims to assess the antibacterial activity and shear bond strength (SBS) of traditional orthodontic adhesives that have been admixed with varying quantities of *C. gigantea* NPs and to compare the results with conventional adhesives that have not been modified.

## Materials and methods

This study was conducted at the Narayana Dental College (Nellore, Andhra Pradesh, India) in collaboration with Shri Vishnu College of Pharmacy (Bhimavaram, Andhra Pradesh, India) and Virtue Meta-Sol laboratories (Hyderabad, Telangana, India). This in vitro study protocol received approval from the Institutional Review Board of Narayana Dental College (approval no. ICE/NDCH/2019/P-39).

Sample size

The sample size calculation was done using GPower version 3.1.9.4 (Bonn University, Bonn, Germany). A minimum total sample of 40 was required with a power of 0.8, an error of 5%, and a confidence interval (CI) of 95% to determine a size difference of 1mm of antibacterial zone between any two samples and 1 MPa of SBS between any two samples. The probability of a p-value <0.05 was considered statistically significant.

Collection of the sample and inclusion/exclusion criteria

Forty maxillary and mandibular premolar teeth, extracted for orthodontic purposes over six months (February 2021 to July 2021), were collected and preserved in thymol solution (0.1%). Sound human premolar teeth therapeutically extracted for orthodontic purposes are included in this study, while teeth with caries, fillings, endodontic treatment, enamel hypoplasia, and enamel cracks were excluded.

Grouping of the sample

The total sample (40) was equally assigned to four groups of 10 each. There were three experimental groups depending on the concentration (1%, 5%, and 10%) of *C. gigantea* NPs admixed in the adhesive system and one control group that did not contain any NPs. Group I, the control group (n = 10), had unmodified conventional adhesive. Group II, the test group (n = 10), received conventional adhesive admixed with 1% *C. gigantea *NPs. The Group III test group (n = 10) got conventional adhesive admixed with 5% *C. gigantea* NPs. And the Group IV test group (n = 10) was allocated conventional adhesive admixed with 10% *C. gigantea* NPs. 

Procedure

**Figure 1:**
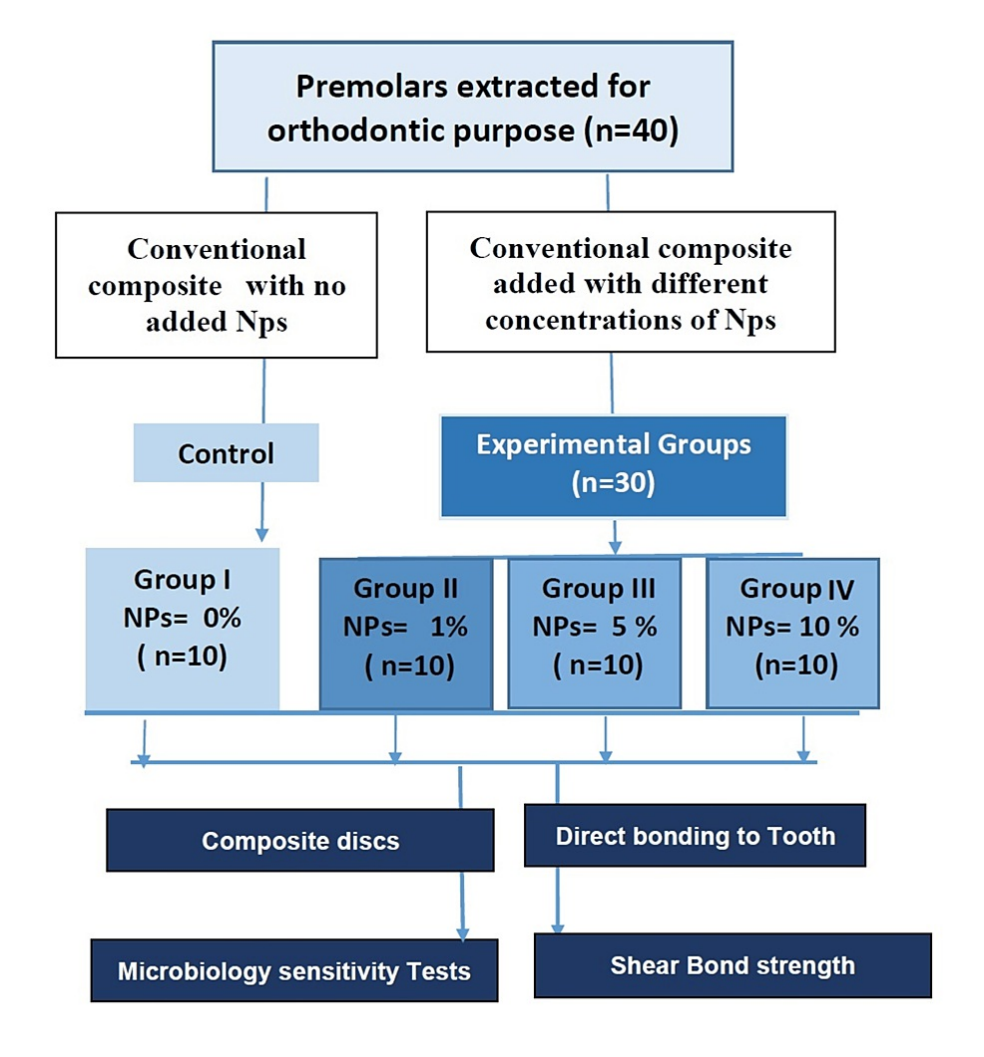
Flow chart showing the experimental setup NPs: Nanoparticles

**Figure 2:**
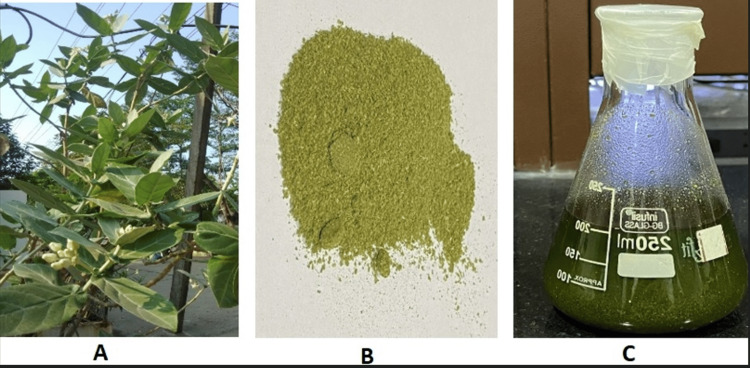
Preparation of liquid extract from the leaves of C. gigantea A: *C. gigantea* plant; B: Powdered form of *C. gigantea* leaves; C: Ethanolic extract of *C. gigantea* leaves

The methodology followed in this study is based on previously published data (Figure 1) [1,10,15-17], while the procedure is adapted from previous studies [12,15]. The process began with the collection of fresh leaves from the *C. gigantea* plant. After thorough washing in running water, the leaves were air-dried for 48 hours in natural light and then finely powdered using a blender. Twenty grams of this powder was dissolved in 200 mL of ethyl alcohol. The resulting solution, at room temperature, underwent stirring for seven days. Then it was filtered through filter papers to eliminate impurities. The solvent (ethanol) was removed from the plant extract to yield a dry product, achieved through the use of a Heidolph rotary evaporator under vacuum conditions with the following parameters: 100 rpm, temperature 55°C, vacuum 5 Torr. The concentrated extract obtained was collected and stored in a desiccator for future use (Figure 2).

Preparation of the *C. gigantea* extract

*Calotropis gigantea* solid lipid nanoparticles (CG-SLNs) loaded with the plant extract (*C. gigantea*) were prepared using the solvent emulsion evaporation technique (SEE) by Pooja et al. [16], with slight modifications. A precise amount (50 mg) of lipid (glyceryl monostearate (GMS)) was placed in a small beaker, and 25 mg of the plant extract was added. These components were solubilized in 3 mL of methanol with stirring to create the organic phase. Simultaneously, in another beaker, 0.1 mL of Tween 80 (1% W/V) was combined with 10 mL of triple-distilled water and heated to 65°C. The lipid mixture was gradually added to the heated aqueous phase under high-speed mixing using an ULTRA-TURRAX® homogenizer (IKA 25 digital, Germany, 2011) at 5000 rpm for five minutes. Mixing continued for an additional five minutes in an ice-cold water bath, followed by allowing the preparation to cool down. The solid NP suspension was then collected in Eppendorf centrifuge tubes (2 mL capacity) and centrifuged at 3000 rpm for 15 minutes. The supernatant was collected and diluted with distilled water for particle size analysis using dynamic light scattering equipment (Horiba SZ-100, HORIBA Advanced Techno Co., Ltd., Kyoto, Japan, 2019).

Characterization of SLN

**Figure 3:**
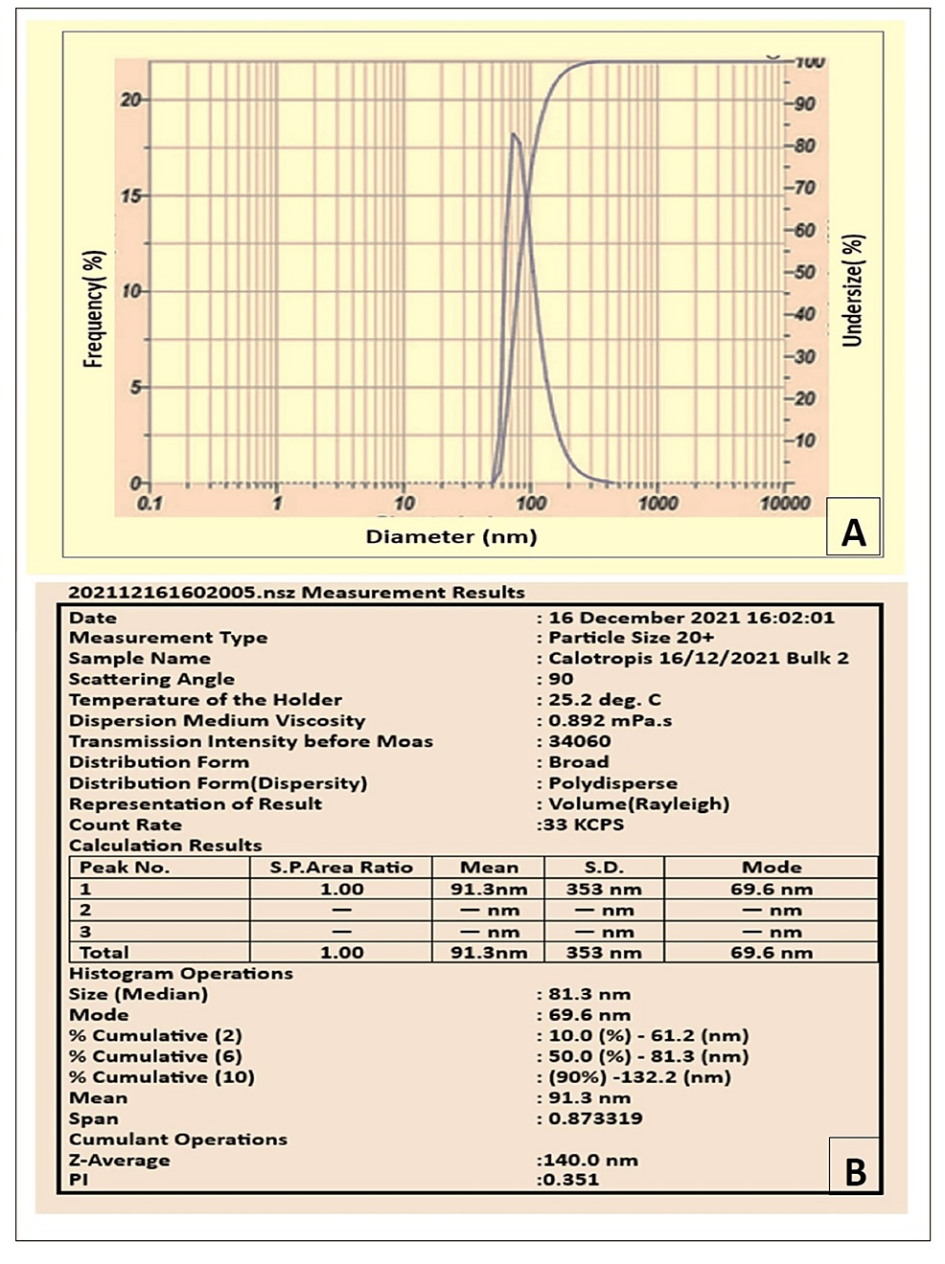
(A) Graph showing the PS of C. gigantea NPs evaluated using Horiba SZ-100; (B) The PDI of the C. gigantea NPs PS: Particle size, NPs: Nanoparticles, PDI: Poly dispersity index

The particle size (PS) and polydispersity Index (PDI) of the NPs were assessed using the Horiba SZ-100 particle size analyzer, as outlined in the previous study [17]. Before analysis, distilled water was used to suitably dilute the material (Figure 3).

Nanocomposite preparation with different concentrations

The admixing procedure was similar to that of a study by Sodagar et al. [1]. In an earlier study [15], the *C. gigantea* mouthwash was shown to exhibit antimicrobial activity at a minimum concentration of 1.5% and also showed that the activity increased with an increase in concentration. Hence, in our study, we have taken a minimum 1% concentration as a basic reference and an additional 5% and 10% to assess the effect of increased concentrations on the antimicrobial properties and SBS. In the initial step, 2700 mg of composite was mixed manually with 300 mg of *C. gigantea* NP powder, yielding a 3000 mg composite with a 10% *C. gigantea* NP concentration. This base-admixed composite was used further to create composites with varying concentrations of *C. gigantea* NPs. For the composite with 5% *C. gigantea* NPs, 900 mg of the 10% composite was combined with 900 mg of the unmixed composite. Similarly, for the composite with 1% C. gigantea NPs, 180 mg of the 10% *C. gigantea* NPs was blended with 1620 mg of plain composite. This resulted in 1920 mg of composite with 10% C. gigantea NPs, 1800 mg of composite with 5% *C. gigantea* NPs, and 1800 mg of composite with 1%* C. gigantea* NPs. The weighing of *C. gigantea* NP powder was performed using a digital scale, and manual mixing of the composite was carried out with a spatula on a glass slab in a light-proof environment to achieve a consistent concentration.

Parameters

Antimicrobial Tests

**Figure 4:**
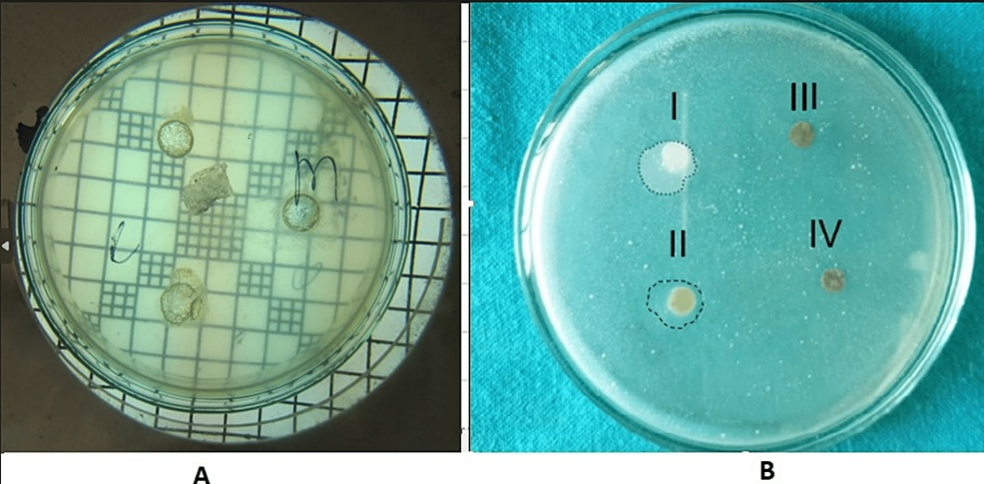
Antimicrobial tests A: Agar well test, where the Petri plate shows no bacterial growth around the wells with *C. gigantea* NPs; B: Agar diffusuion disc test shows the inhibition zones extending around the Group I (unmodified) and Group II (1% NP) composite discs NP: Nanoparticles, I: Group I (0%NP), II: Group II (1% NP), III: Group III (5% NP), IV: Group IV (10% NP)

Agar well diffusion test: Around 10 mg of the prepared nanoparticles were dispersed in 2 ml of distilled water. A mixed bacterial culture was then inoculated into sterile agar media, which was then poured into Petri plates to create the plates. Subsequently, 50 microliters of the *C. gigantea *NP suspension were carefully deposited into the wells of the Petri plate (Figure 4) and incubated at 37°C for 24 hours.

Disc agar diffusion (DAD) test: The antimicrobial activity of the discs prepared from the composite material of all the groups was assessed. This test assessed the ability to dissolve and diffuse *C. gigantea* NPs. This assay evaluated the *C. gigantea *NPs' dissolution and diffusion abilities. Ten discs of 1 mm thickness and 6 mm diameter for each adhesive group prepared by silicone molds were utilized for this test.

Bacterial suspensions, including *S. mutans* ATCC25175 and* L. acidophilus* ATCC4356 obtained in lyophilized form, were rehydrated and incubated in broth for 48 hours under anaerobic conditions at 37°C. Microorganism suspensions with a concentration of 108 CFU/ml were prepared using a spectrophotometer. A bacterial suspension is produced by using swabs and then cultured evenly on Mueller Hinton medium supplemented with 5% sheep blood. Subsequently, composite discs from each group were positioned on the medium-containing plates (Figure 4). A carbon dioxide (CO2) incubator was used to keep all of the culture-rich medium plates at 37˚C for a whole day. Following incubation, the diameter (mm) of the inhibitory zone of the growth of bacteria surrounding the discs was measured using an electronic ruler.

Mechanical shear bond strength test

Bonding of the Brackets

**Figure 5:**
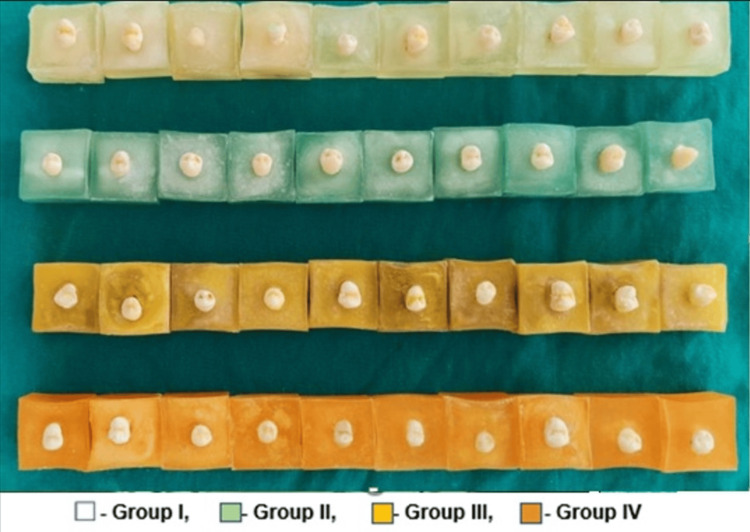
Premolar teeth embedded in color-coded acrylic blocks for shear bond test NP: Nanoparticle, Group I: 0%NP), Group II: 1% NP; Group III: 5% NP; Group IV: 10% NP

The 40 selected sample teeth were randomly distributed among four groups to ensure an equal sample distribution of n = 10 each. Each tooth was then embedded in a colored acrylic block (1×1×1 inch), exposing only the coronal portion of the specimen (Figure 5). The crown portions of the teeth were oriented along the vertical axis of the acrylic blocks. After cleaning the buccal surfaces with a prophylaxis brush, washing with water, and drying, the teeth underwent 30 seconds of etching with 37% phosphoric acid. Subsequently, they were rinsed and dried with an air spray for 15 seconds. A thin layer of primer (Transbond XT primer) was then applied to the center of the tooth on the buccal side and light-cured for 10 seconds each. Brackets were bonded using adhesives assigned to each group. Bondable stainless steel 0.022” x 0.028” slot MBT premolar brackets (Ormco Mini Diamond®, Ormco, Brea, CA, USA) were utilized, with the bracket base having an approximate surface area of 12.62 mm2. The composite was light-cured for 10 seconds from each aspect for a total of 40 seconds using an LED curing unit (Woodpecker Light Cure LED Mini-S, Equitech Engineers Pvt. Ltd., Pune, MH, India) after removing excessive adhesive flash. All specimens underwent 1000 rounds of thermocycling in a thermocycling chamber (Termotron S-8C, Thermotron Industries, Holland, MI, USA) within 24 hours to simulate the environment of the oral cavity. Each cycle comprised a 15-second immersion in a 5°C water bath, a 10-second dwell time, and a 15-second immersion in a 55°C water bath.

**Figure 6:**
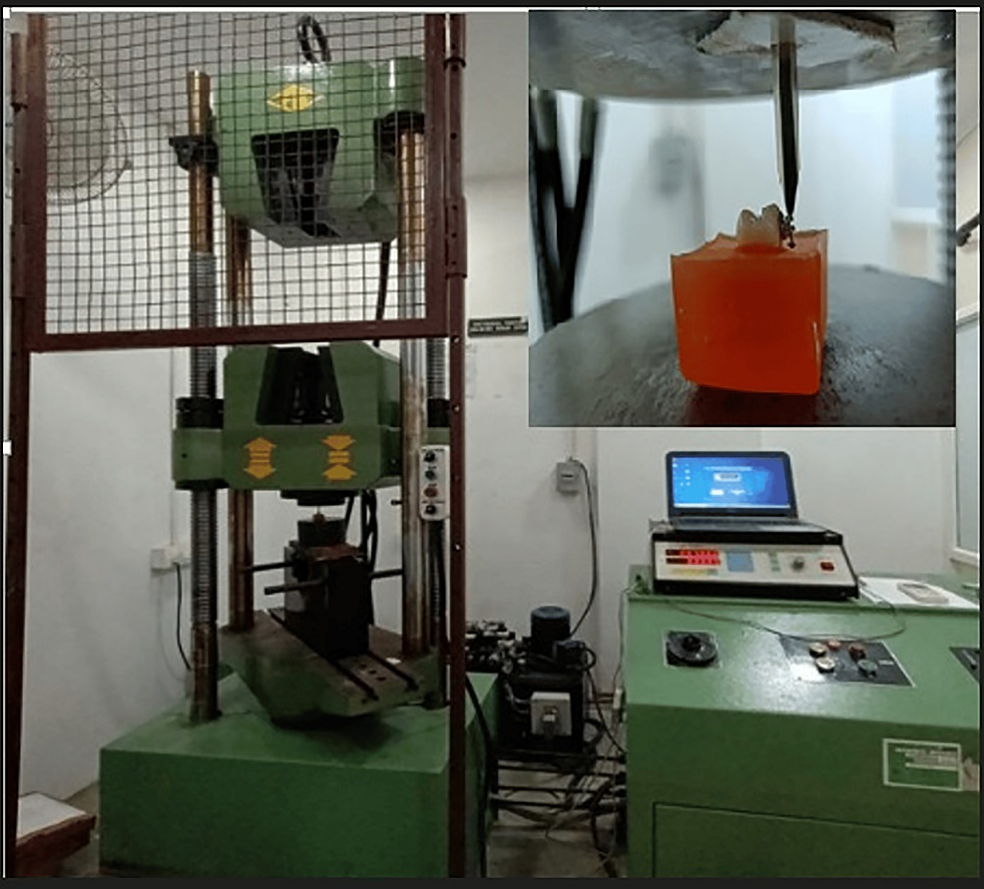
Universal Testing Machine(FIE-UTES-40-HGF) and the inset showing the setup for the SBS measurement of the bonded brackets SBS: Shear bond strength

Following thermocycling, the brackets received shear forces from an electronic universal testing machine (FIE-UTES-40-HGF, Fuel Instruments & Engineers Pvt. Ltd., Kolhapur, MH, India) (Figure 6). Specimens were positioned with the bracket base aligned parallel to the direction of force applied. Shear forces were applied at the composite interface in an occluso-cervical direction by a metal blade of 0.6 mm diameter with a speed of 0.5 mm/min until the brackets were debonded. The SBS in MPa was derived from the magnitude of force value (N) divided by the area of the bracket base.

Data collection and statistical analysis

All the parameters are measured as continuous data, and the average mean and SD for each group were calculated. Microsoft Excel (Microsoft Corp., Redmond, WA, USA) was utilized for the entry of the collected data and statistical analysis using SPSS Statistics version 27 (IBM Corp., Armonk, NY, USA). The normality of mean values for SBS and microbial inhibition zone diameter was assessed using the Shapiro-Wilk test. Given that the data for SBS and microbial inhibition zone diameter are continuous, quantitative, and met the parametric assumptions, a one-way ANOVA was employed as an inferential statistical test to analyze differences among the four groups. Tukey’s honestly significant difference (HSD) post-hoc test facilitated intra-pair group comparisons. A significance probability (p) value of 0.05 or less was considered for all analytical tests, indicating statistical significance in the differences between any two groups.

## Results

The mean values for the diameter of the inhibition zone and SWBS are given in Table 1 and Table 2.

**Table 1:** Descriptive data of microbial inhibition zone diameter (mm) NP: Nanoparticle

Microorganism	Group (NP%)	n	Minimum	Maximum	Mean	SD
S. mutans	Group I (0%)	10	9.5	12.1	11.09	0.87
	Group II (1%)	10	8	10.1	8.92	0.67
	Group III (5%)	10	0	0	0	0
	Group IV (10%)	10	0	0	0	0
L. acidophilus	Group I (0%)	10	9.3	11.9	10.80	0.78
	Group II (1%)	10	8.1	9.9	8.9	0.58
	Group III (5%)	10	0	0	0	0
	Group IV (10%)	10	0	0	0	0

**Table 2:** Descriptive data of SBS (MPa) in the four groups NP: Nanoparticle, SBS: Shear bond strength

Group	n	Minimum	Maximum	Mean	SD
Group I (Control 0% NP)	10	6.09	8.72	6.99	0.75
Group II(1% NP)	10	5.48	7.84	6.29	0.67
Group III (5% NP)	10	3.84	5.49	4.40	0.47
Group IV (10% NP)	10	1.73	2.47	1.98	0.21

The particle size analyzer determined that the prepared NPs had an average PS of 140 nm and a PDI of 0.351 (Figure 3). The results of the agar well diffusion test indicated no bacterial growth in Petri plates inoculated with the *C. gigantea* NP suspension (Figure 4), demonstrating significant inhibition of bacterial growth compared to plates without *C. gigantea *NPs. The DAD test to assess the microbial inhibition zone diameter data for *S. mutans* and *L. acidophilus* in different concentration groups (Figure 4, Table 3), showed a significant difference in bacterial inhibition among groups (p < 0.001). Intrapair differences are statistically significant for all combinations of the pair tested (p < 0.001). Incorporating *C. gigantea* NPs decreased the microbial potency of the conventional adhesive (Table 4).

**Table 3:** Intergroup and intragroup comparisons and one-way ANOVA of inhibition zone diameter (mm) of the four groups for S. mutans and L. acidophilus *p < 0.05 is considered statistically  significant

Bacteria	Source	Sum of squares	Degree of freedom	Mean square	F-ratio	p-value
S. mutans	Between groups	1230.1675	3	410.0558	1355.38	< 0.001*
	Within groups	13.3117	44	0.3025		
	Total	1243.4792	47			
L. acidophilus	Between groups	1187.1056	3	395.7019	1644.21	< 0.001*
	Within groups	10.5892	44	0.2407		
	Total	1197.6948	47			

**Table 4:** Intrapair comparison and Tukey’s HSD post hoc test of inhibition zone diameter (mm) of the four groups for S. mutans and L. acidophilus *p < 0.05 is considered statistically significant HSD: Honestly significant difference

S. mutans		HSD_.05_ = 0.5996 HSD_.01_ = 0.7411	Q_.05_ = 3.7760 Q_.01_ = 4.6673	p-value
Pairwise comparison				
G_1_:G_2_	M_1_ =11.09;M_2_ = 8.93	2.17	Q = 13.65	< 0.001*
G_1_:G_3_	M_1_ =11.09;M_3_ = 0.00	11.09	Q = 69.86	< 0.001*
G_1_:G_4_	M_1_ =11.09;M_4_ = 0.00	11.09	Q = 69.86	< 0.001*
G_2_:G_3_	M_2_ = 8.93;M_3_ = 0.00	8.93	Q = 56.21	< 0.001*
G_2_:G_4_	M_2_ = 8.93;M_4_ = 0.00	8.93	Q = 56.21	< 0.001*
G_3_:G_4_	M_3_ = 0.00;M_4_ = 0.00	0.00	Q = 0.00	< 0.001*
L. acidophilus		HSD_.05_ = 0.5347 HSD_.01_ = 0.6610	Q_.05_ = 3.7760 Q_.01_ = 4.6673	p-value
Pairwise comparison				
G_1_:G_2_	M_1_ =10.81;M_2_ = 8.90	1.91	Q = 13.48	< 0.001*
G_1_:G_3_	M_1_ =10.81;M_3_ = 0.00	10.81	Q = 76.32	< 0.001*
G_1_:G_4_	M_1_ =10.81;M_4_ = 0.00	10.81	Q = 76.32	< 0.001*
G_2_:G_3_	M_2_ = 8.90;M_3_ = 0.00	8.90	Q = 62.85	< 0.001*
G_2_:G_4_	M_2_ = 8.90;M_4_ = 0.00	8.90	Q = 62.85	< 0.001*
G_3_:G_4_	M_3_ = 0.00;M_4_ = 0.00	0.00	Q = 0.00	< 0.001*

The SBS test showed that the control group had the highest mean SBS (6.994±0.75 MPa), while the 10% NP concentration group had the lowest (1.984±0.21 MPa) (Table 1). A one-way ANOVA indicated a significant difference in SBS among groups (p < 0.001). Intrapair differences are significant between all groups except between the SBS values of the control group and the 1%* C. gigantea* NPs group (p = 0.04), both falling within the clinically acceptable range (5.9-7.8 MPa) (Table 5).

**Table 5:** Intergroup and intragroup comparisons of SBS values (Mpa) of the four groups using one-way ANOVA, and pairwise comparisons using Tukey’s HSD post hoc test *p < 0.05 is statistically significant Superscript a b c d : Those without a common superscript alphabet indicate significant difference (p <0.05) compared to other groups  across the rows (pairwise comparison using Tukey’s HSD post hoc test) B/G: Between groups, W/G: Within groups, SBS: Shear bond strength, HSD: Honestly significant differe Superscript^ a b c d ^: Those without a common superscript alphabet indicate significant difference (p <0.05) compared to other groups  across the rows (pairwise comparison using Tukey’s HSD post hoc test)

Groups	(n)	Mean	Sum of squares	Degrees of freedom	Mean square	F-ratio	p-value
Group I (0% NP)	10	6.99^ab^	B/G-150.78 W/G-11.61 Total-162.39	B/G-3 W/G-36 Total-39	B/G-50.26 W/G-0.32	155.82	< 0.001*
Group II (1% NP)	10	6.29^ba^					
Group III (5% NP)	10	4.40^c^					
Group IV (10% NP)	10	1.98^d^					

## Discussion

Several methods to eliminate or reduce demineralization of the enamel surface that results in the formation of WSLs during orthodontic treatment mostly rely on patient compliance [4]. One such effective method for preventing enamel demineralization that doesn't depend on patient compliance is the use of orthodontic adhesives with antibacterial properties, which make them resistant to the adhesion of bacteria and biofilm formation [6-12]. Among the materials tested for antimicrobial activity, metallic synthetic NPs have received a lot of attention because of their bactericidal and plaque inhibition properties and target delivery. However, there is a concurrent risk of cytotoxicity in cells due to the release of metal ions, according to a few studies [18,19].

Large concentrations of antimicrobial compounds can be found in medicinal plants, making them a valuable resource. Since ancient times, there has been a growing curiosity and interest in the use of plant extracts and other biologically active substances that are isolated from these plants to cure a variety of ailments [10,11,13]. Therefore, using the NPs synthesized from natural products, especially plant extracts, would be beneficial. In dentistry, especially in orthodontics, few studies [10,11] have been conducted using plant extracts. The studies of Sodagar et al. established that orthodontic adhesives modified with curcumin NPs (curcNPs) at 1 weight% have anti-activity against cariogenic bacteria [10], and nanopropolis (prpNPs) at 2% and 5% concentrations have a profound antibacterial effect [11].

*Calotropis gigantea* is one such plant that is well-known for its medicinal properties. A deep look into the literature revealed that only two studies have been conducted on this plant in the dental field [15,20], and no such studies have been conducted so far using this plant from the perspective of orthodontics. According to the findings of the in vivo study [20], *C. gigantea*, when used as a mouth rinse, has antimicrobial efficacy and was found to be effective against *S. mutans* and *Lactobacillus casei*. Considering the current interest in the antimicrobial activity of the *C. gigantea *plant and the gap in evidence regarding their antimicrobial efficacy, we pursued this study using this plant to assess its antimicrobial efficacy when used in NP form admixed with orthodontic adhesive. This was the first study in the orthodontics field using *C. gigantea* NPs in orthodontic adhesives. In the current study, we synthesized NPs using this plant extract, as NPs are highly effective in producing antimicrobial properties because of their reduced size and high membrane-penetrating ability [14].

Using the manual mixing method, the *C. gigantea* nanocomposite of different concentrations (1%, 5%, and 10%) was prepared, and antimicrobial activity was assessed against *S. mutans* and *L. acidophilus*. These two bacterial strains are utilized in this study, as they are primarily involved in the demineralization of the tooth and WSL formation. Caries is initiated primarily by the action of *S. mutans* while the progression of caries occurs through the activity of L. acidophilus [2,21].

The size of the nanoparticles formed in this study is 140 nm, with a PDI of 0.35 (Figure 3). Controlling and validating the parameters of nanocarrier formulations' average diameter, PDI, and size stability is very important in deciding the suitability of a specific mode of delivery [22]. The PDI values in this study indicate that there is uniformity in the size distribution.

Antimicrobial activity was assessed by the agar well diffusion and DAD tests. The agar well diffusion test indicated the ability of *C. gigantea* NPs to inhibit the growth of bacteria. The DAD test provides the extension zone of activity of the antibiotic under test. In the context of orthodontic adhesive, this test aptly measures the ability of the antimicrobial adhesive to control WSLs that form at the bracket-enamel surface interface. This test is important to determine the ideal antimicrobial agent added to the orthodontic adhesive that can diffuse into the surrounding medium since WSLs are normally formed around the bracket base rather than underneath [23].

The present study showed that the composite disks of the control group and the 1% *C. gigantea *NP group formed a growth inhibition zone against *S. mutans *and *L. acidophilus*. However, the growth inhibition zone of the discs made of 1% *C. gigantea *NPs (mean 8.92 mm for S. mutans and 8.9 mm for L. acidophilus) was lower compared to that of the control group (mean 11.09 mm for *S. mutans* and 10.8 mm for *L. acidophilus*) and is statistically significant (p < 0.01). In the clinical setting, the maximum exposed tooth surface around the brackets is <5 mm on all sides. The inhibition zone diameter achieved with 1% *C. gigantea* NPs is within these bounds and therefore may still be effective against the cariogenic bacteria on the tooth surface around the brackets. No inhibition zone of growth was seen around discs of 5% and 10%* C. gigantea* NP concentration groups for both the bacteria tested. This indicates insolubility and poor diffusion of* C. gigantea *NPs into the medium around composite discs (Tables 1, 3-4).

The results obtained in the current study can be compared to the few studies that incorporated metallic adhesives as organic NPs. Earlier studies [1,9,24,25] with various metallic NPs incorporating adhesives, irrespective of their concentration in the orthodontic adhesives, displayed antimicrobial efficacy. Eslamian et al. [24] reported that silver (Ag) NPs, when used at 0.3% weight concentration, showed a significant antibacterial effect against *S. mutans* at both time points (24 hours and 30 days) when compared with that of the control group. Similarly, Sodagar et al. [1] showed that titanium dioxide (TiO2) NPs of all concentrations (1%, 5%, and 10%) showed significant antimicrobial effects against *S. mutans*,* L. acidophilus*, and* Streptococcus sanguinis* compared to the control group, and the inhibition zone was highest for the 10% TiO2 nanoparticle group. Likewise, Toodehzaeim et al. [25] also reported that copper oxide (CuO) NPs of all the concentrations (0.01%, 0.5%, and 1%) after 48 hours of incubation showed microbial inhibition zones when tested against *S. mutans*, and the maximum zone of growth inhibition for *S. mutans* with a mean diameter of 9.20mm belonged to adhesives with 1% weight of CuO NPs. The antibacterial activity was shown to be directly proportional to the concentration of NPs. A study by Yassaei et al. [9], however, concluded that CuO and 1% silver oxide NPs provide only short-term antibacterial effects and do not have long-term effects as measured by colony counts of* S.mutans *at the end of 30 days compared to controls.

Some of the previous studies utilized biological NPs at various concentrations. One such study is by Sodagar et al. [11], which reported that prpNPs after 24 hours of incubation had a significant antimicrobial efficacy against *S. mutans *and *S. sanguinis *at 2%, 5%, and 10% concentrations, but no bacterial inhibition zone was seen for discs containing 1% prpNPs. Also, no inhibition zone against *L. acidophilus *was formed for all the concentrations tested.

In contrast, the findings of Sodagar et al. [10] on the addition of curcNPs (*Streptococcus*) are similar to our study. They observed no growth inhibition zones in any one of the groups with 1%, 5%, and 10% concentrations. However, a significant reduction of colony counts after 30 days was observed by an increase in the percentage of curcNPs and a maximum reduction of colony counts with 10% concentrations. They attributed this contrast feature to the poor solubility and diffusion of curcumin particles despite strong bactericidal activity. They concluded that adhesives admixed with 1% curcNP concentrations exhibited significant anticariogenic activity against bacteria without compromising SBS. However, the main drawback of curcNPs is their poor solubility and diffusion. Similarly, Pourhajibagher et al. [12] in their DAD assay at 24 hours of incubation showed that photo-activated cCur/zinc oxide (ZnO)NPs at 7.5% weight basis had antimicrobial activity towards cariogenic bacteria such as *L. acidophilus*, *S. mutans*, and *Streptococcus sobrinus*, and up to a 60-day experimental period thereafter, which it reduced. The other concentrations below and above 7.5% Cur/ZnONPs showed antimicrobial activity. Thus, from the above studies, it can be noticed that microbial inhibition seems to be dependent on the type of NP used and the periods at which it was measured rather than the incubation time and concentration of NPs used. Further studies need to be explored in the future regarding this parameter in this field. In the current study, isolated *C. gigantea* NPs showed antimicrobial efficacy when tested alone. But when mixed with the composite, it failed to show its antimicrobial efficacy, and instead, the antimicrobial activity of the adhesive (Transbond XT) used in this study was found to be decreased. This might be due to the inability or non-diffusion of NPs in the medium. The diffusion capacity of *C. gigantea* NPs needs to be investigated in further studies.

The SBS between the bracket and enamel should be sufficient to resist the dislodgement forces delivered during function as well as by the treatment procedures. Several studies [1,10,12,24] have reported that the incorporation of antimicrobial agents into composites affects the chemical nature of the adhesives and decreases the adhesive bonding forces at the bracket-enamel surface interface. The present study also evaluated the bond strength against shearing forces after the composites were admixed with *C. gigantea* NPs. The results revealed that there is a significant reduction in SBS with the increase in the percentage concentration of *C. gigantea* NPs in the adhesive (Table 2, Table 5). The maximum values of SBS belonged to the control group (6.99±0.75 MPa), while the lowest mean value was for the 10% NP composite group (1.98±0.21 MPa). The control or unmixed (6.99 MPa) and the composite containing 1% *C. gigantea* NPs (6.296 MPa) values of both groups were the only ones within the acceptable clinical range of 5.9-7.8 MPa [26]. The present values of SBS are concordant with the studies that utilized nonmetallic NPs with the other experimental adhesives admixed with curcumin [10] and prpNPs [11]. The findings are similar in that there is a dose-dependent decrease in the SBS with an increased proportion of organic NPs. A similar trend was also seen with ZnO NPs cationic doped with curcumin (cCur/ZnONPs) [12].

The SBS values of all the concentrations of 1%, 5%, and 10% with curcumin-incorporated adhesives were well above the clinically acceptable range. There are contrary results from the existing studies that tested the metallic NPs. The studies utilizing TIO2 [1] and Ag+ [24] exhibited a reduction in bond strength when compared with their respective control groups with increasing concentrations of NPs, which was in line with the present study. C

ontrary to the results of the present study, the studies that included the CuO NPs [8,25] displayed an increase in the SBS with the increase in the concentration of the particles. Argueta-Figueroa et al. [8] in their study showed that the addition of Cu NPs to the adhesive at 0.01 weight% (15.2 MPa) instead increased the SBS compared to the control group (9.5 MPa). Similarly, Felemban and Ebrahim [27] also reported that composites with zirconium oxide and titanium dioxide (ZrO2-TiO2) NPs increased the SBS at both 0.5 weight% (20.32 MPa) and 1 weight% (25.05 MPa) concentrations. Similarly, Akhavan et al. [28] found that the SBS increased because of the addition of Ag and hydroxyapatite NPs to the composite at 1% (5.72 MPa) and 5% (8.16 MPa) concentrations, respectively, which is similar to the findings of the current study. Poosti et al. [7], in their study with 1% TiO2 concluded that addition of NPs couldn’t compromise the SBS and debonding patterns of adhesives.

This difference in the magnitude of SBS from various studies could be mainly due to the different types of NPs tested as well as the thermocycling processes and methods of mixing the NPs. In the present study, less bond strength was observed for the control group compared to the previous studies. The decrease in SBS might be due to the darker adhesive colors and less translucency caused by an increase in some nanoparticle concentrations, which hinder full adhesive curing by reducing the depth of light penetration. Inadequate curing thereby leads to a decrease in SBS [28]. The other reason for the reduced SBS in our study might be due to the thermocycling procedure, which, according to a few studies [8,27], caused a significant decrease in the SBS. We have manually mixed the nanocomposites using the spatula and glass slab; porosities might have been incorporated that can lead to polymerization shrinkage, which in turn leads to reduced SBS. The null hypothesis in the present study stands rejected as a significant difference was seen in the antimicrobial efficacy and SBS between conventional orthodontic adhesive modified with *C. gigantea* NPs and non-admixed conventional adhesive.

No study goes without limitations. As such, in our study, only the DAD test was used for determining the antimicrobial activity; it would be more advantageous to use other antimicrobial tests. The cytotoxicity of *C. gigantea *NPs and adhesive remnant index (ARI) scores were also not evaluated in the current study. The color of the orthodontic adhesive admixed with *C. gigantea* Nps is another major drawback of this study, as it is not only aesthetically unacceptable but also inhibits polymerization because of its darker shade.

Clinical implications of the study

In this study, though orthodontic adhesive admixed with *C. gigantea *NPs showed no additive antimicrobial properties compared to non-admixed conventional adhesive, *C. gigantea* NPs alone proved to have antimicrobial efficacy. Green synthesis of metal NPs using extracts of *C. gigantea* can be performed to increase the diffusing capacity and hence increase the effective anticariogenic activity of *C. gigantea* NPs. These NPs can be used in other dental and orthodontic materials where the color of the material is not a priority, as the *C. gigantea* NPs produce darker shades at high concentrations in orthodontic adhesives. If at all to be used in adhesives, *C. gigantea* NPs in lower concentrations might be effective. As this was the first study to use the *C. gigantea *NPs, further studies are recommended using the *C. gigantea* NPs regarding their antimicrobial efficacy, cytotoxicity, and molecular interactions when incorporated into various orthodontic materials like adhesives, primers, and coatings.

## Conclusions

Freshly prepared *C. gigantea *NPs, when used alone, exhibited antimicrobial activity. Increasing the percentage concentration of the NPs decreased the microbial activity as well as the bond strength. The SBS values of the 1% C. gigantea NPs group have clinically acceptable bond strengths with antimicrobial activity.
